# Gut microbiota‐derived butyrate mediates the anticolitic effect of indigo supplementation through regulating CD4^+^ T cell differentiation

**DOI:** 10.1002/imt2.70040

**Published:** 2025-04-19

**Authors:** Yunqi Xing, Muyuan Wang, Yali Yuan, Jiayan Hu, Zhibin Wang, Zhongmei Sun, Mengyu Zheng, Lei Shi, Junxiang Li, Tangyou Mao

**Affiliations:** ^1^ Dongfang Hospital Beijing University of Chinese Medicine Beijing P.R. China; ^2^ Yueyang Hospital of Integrated Traditional Chinese and Western Medicine Shanghai University of Traditional Chinese Medicine Shanghai P.R. China; ^3^ Tianjin Nankai Hospital Tianjin P.R. China; ^4^ King's College London UK

## Abstract

This study explored the effect of plant‐derived indigo supplementation on intestinal inflammation using in vivo, in vitro, and clinical sample analyses. Our results showed that indigo decreased mucosal inflammation by regulating CD4^+^ T cell differentiation in a gut microbiota‐dependent manner. Microbes transferred from indigo‐treated mice, indigo‐induced enrichment of *Roseburia intestinalis*, and its metabolite butyrate played a role in Th17/Treg immunity similar to that of indigo in intestinal inflammation, which was involved in mTORC1/HIF‐1α signal‐mediated reprogrammed glucose metabolism. We further showed that patients with ulcerative colitis exhibited significant gut dysbiosis and CD4^+^ T cell differentiation abnormalities. Our findings provide new insights into the gut‐immune axis in ulcerative colitis, offering a novel microbial‐based immunotherapy for the treatment of inflammatory bowel disease.

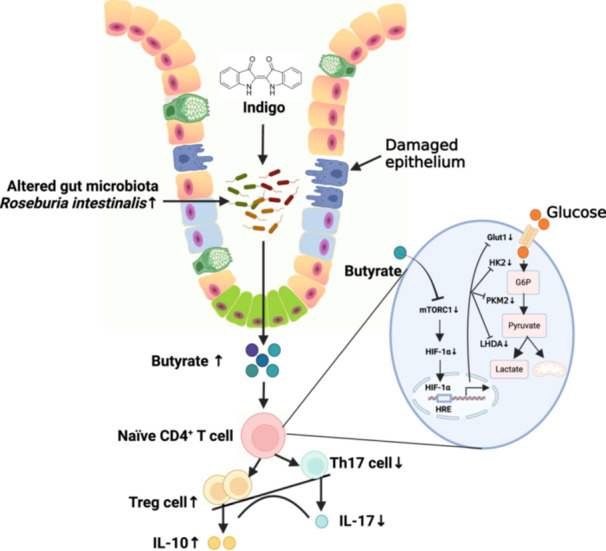

Ulcerative colitis (UC) is a chronic and recurrent inflammatory intestinal disorder characterized by abnormal mucosal immunity. Current therapies, including aminosalicylic acid, steroid hormones, immunosuppressants, and small‐molecule drugs, focus on inducing and maintaining disease remission [1, 2]. These approaches can achieve certain therapeutic effects in the short term but have several limitations, such as low responsiveness, opportunistic infections, and high recurrence [3, 4]. Therefore, there is an urgent need to develop therapeutic agents that can halt or prevent UC progression.

The human gastrointestinal tract is a complex and dynamic ecosystem that harbors thousands of different coevolved microorganisms that contribute to the maintenance of the epithelial barrier and homeostasis of the host immune system [5]. The complex and mutualistic interactions between mucosal immunity and the gut microbiota provide resistance to colonization by enteropathogens and help maintain physical health [6]. Emerging evidence indicates that the gut microbiota and its metabolites are essential for T cell differentiation and activation and maintenance of the Th17/Treg immune balance [7]. If the crosstalk between the gut microbiota and immunity goes awry, it will disrupt the intestinal host immune responses and alter the intestinal barrier, leading to an exaggerated inflammatory response in the intestine [8]. Therefore, targeting the interaction between the gut microbiota and intestinal immune system is the main strategy for preventing and treating UC.

Indigo, a pharmacologically active component of indigo naturalis, exhibits multiple anti‐inflammatory activities. However, its effects and underlying mechanisms, especially on the complex interface between the intestinal epithelium, microbiota, and mucosal immunity in UC, remain elusive. Our results show that indigo alleviates intestinal inflammation through gut microbiota‐derived butyrate‐mediated immunity homeostasis reconstruction via the modulation of CD4^+^ T cell differentiation and holds promise as a novel microbial‐based immunotherapy for UC treatment.

## RESULTS AND DISCUSSION

### Administration of indigo ameliorated intestinal inflammation and improved Th17/Treg cell balance in colitis mice

**Figure 1:**
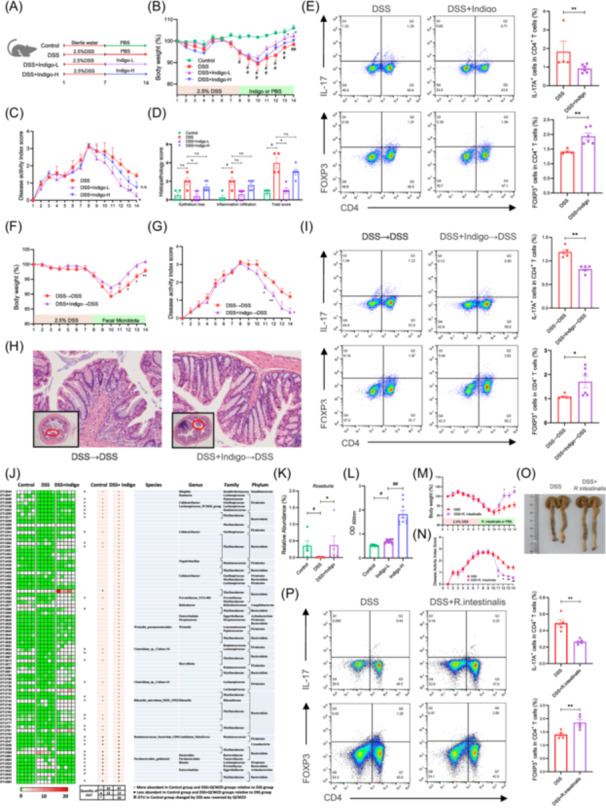
Indigo alleviates intestinal inflammation by regulating Th17/Treg balance in a gut microbiota‐dependent manner. C57BL/6 mice had free access to sterile water containing 2.5% DSS for 1 week to induce colitis, followed by 7 days of intragastric administration of indigo suspension at a dose of 60 mg/kg and 300 mg/kg or PBS; mesenteric lymph nodes (MLNs) from mice were collected, and the percentages of Th17 and Treg cells were analyzed. (A) Experimental schematic of the pharmacodynamic of indigo. (B) Body weight. (C) Disease activity index (DAI) score. (D) Corresponding histopathological scores of colon tissues. (E) Representative plots and bar charts of the percentage of CD4^+^ IL17^+^ (Th17) and CD4^+^ FOXP3^+^ (Treg) cells in MLNs. Fecal material (stool pellets, and cecal and colonic contents) from donor mice were collected and orally gavaged to the corresponding recipients. Clinical status was assessed throughout the experiment, and MLNs and colon from each mouse were collected for flow analysis and RT‐qPCR. (F) Body weight change. (G) DAI score. (H) Representative H&E staining. (I) CD4^+^ IL17^+^ cells (Th17s) and CD4^+^ FOXP3^+^ cells (Tregs) in MLNs were analyzed by flow cytometry, and the results were displayed as bar charts. Fecal samples from all mice were collected for microbiome profile analysis using bacterial 16S rRNA gene sequencing analysis. (J) Heatmap of abundant OTUs at the species, genus, family, and phylum levels in each group. (K) Relative abundance of *Roseburia* from 16S rRNA gene sequencing analysis. (L) In vitro bacterial cultures of *R. intestinalis* with indigo treatment showed that indigo has a direct and significant growth‐promoting effect on the growth of *R. intestinalis* at 72 h. C57BL/6 mice were administered 2.5% DSS for 1 week, followed by 1 × 10^9^ CFU *Roseburia intestinalis* suspended in 150 μL or PBS per day for 7 days, after which the growth performance and the severity of colitis of all mice were evaluated. (M) Body weight change. (N) DAI score. (O) Colonic morphology. (P) Th17s and Tregs in MLNs were analyzed by flow cytometry, and the results were displayed as bar charts. All data are presented as means ± SEM (*n* = 4–6 per group) from one of three experiments performed showing similar results. ANOVA followed by Tukey's multiple comparison test for B, C, F, G, M, N; Kruskal–Wallis test followed by Dunn's multiple comparisons test for D, K, L; Student's *t*‐test for E (Tregs), P; and Mann–Whitney *U* test for E (Th17s), I. ^##^ *p* < 0.01, ^#^ *p* < 0.05 versus the Control group; ∗∗*p* < 0.01, ∗*p* < 0.05 versus the DSS group; n.s., not significant.

To identify the potential therapeutic role of indigo in intestinal inflammation, a mouse colitis model was established (Figure 1A). Mice treated with indigo, especially at a dose of 60 mg/kg, showed faster recovery from dextran sulfate sodium (DSS)‐induced epithelial injury (Figure 1B–D, Figure S1A–D) and improved Th17/Treg balance (Figure 1E, Figure S1E–J). To further investigate the regulatory mechanisms of indigo in Th17/Treg immune balance, a human‐derived CD4^+^ T cell in vitro culture model was established. Naïve CD4^+^ T cells were sorted from patients with UC to a purity of 99% (Figure S2A). Then, naïve CD4^+^ T cells were cultured under Th17‐ or Treg‐polarizing conditions. The results showed that indigo did not directly affect the differentiation of naïve CD4^+^ T cells into Th17 cells, as evidenced by the lack of significant changes in the number of Th17 cells and IL‐17 levels (Figure S2B–D). Similarly, Treg differentiation was not affected by indigo treatment (Figure S2E–G). These results indicate that indigo does not directly affect the differentiation of naïve CD4^+^ T cells into Th17/Treg cells but acts through an unknown indirect pathway.

### Gut microbiota mediates the immunoprotective effects of indigo treatment on intestinal inflammation in mice

The gut microbiota is essential for T cell differentiation and maintenance of the Th17/Treg immune balance [9, 10]. Therefore, we hypothesized that the gut microbiota may play an important role in the treatment of colitis by indigo. To test this hypothesis, mice were treated with broad‐spectrum antibiotics, which effectively eliminated intestinal bacteria (Figure S3A,B). Consistent with the above protective effects in colitis, indigo treatment resulted in rapid recovery from intestinal inflammation and Th17/Treg imbalance after DSS‐induced mucosal damage. However, depletion of the gut microbiota significantly diminished the beneficial effects of indigo on colitis mice (Figure S3C–L). To further confirm the essential role of the gut microbiota in the treatment of intestinal inflammation by indigo, fecal microbiota transplantation was performed, which showed that colitis mice that received microbiota from indigo‐treated donors experienced less intestinal inflammation and a reconstructed Th17/Treg balance (Figure 1F–I, Figure S4). These findings indicate that indigo protects mice against intestinal inflammation in a gut microbiota–dependent manner and affects the Th17/Treg balance.

To better understand whether and how indigo improved the Treg/Thl7 balance, we examined the gut microbiota composition. Our results showed that indigo increased the number of observed operational taxonomic units (OTUs) and reversed the diversity of the microbiota to a certain extent (Figure S5A–D). Principal components analysis showed that the separation of these community structures was less obvious between the control and DSS+Indigo groups (Figure S5E). At the phylum level, the data showed that indigo altered the Firmicutes‐to‐Bacteroidetes ratio compared to colitis mice (Figure S5F). We further investigated the degree of bacterial taxonomic similarity and revealed that the relative abundance of 80 OTUs changed after the indigo intervention compared with that in the DSS mice, with 67 OTUs increasing and 13 OTUs decreasing. Overall, the relative abundances of 33 OTUs changed after DSS administration and were subsequently reversed after indigo supplementation, maintaining the same trend as that in the control group. Notably, indigo administration enriched *Roseburia* and *Rikenella*, and decreased *Bacteroides* and *Enterorhabdus* (Figure 1J).

### Indigo‐induced enrichment of *Roseburia intestinalis* alleviates intestinal inflammation in mice

To identify the pivotal bacteria functionally involved in the indigo‐mediated immunoprotective effects against colitis, we performed a linear discriminant effect size analysis and showed that *Roseburia* was the marker bacteria of the indigo‐treated mice (Figure S5G). Genus‐level analysis also showed that indigo significantly increased the growth of *Roseburia* (Figure 1J,K). Based on previous studies showing the protective effects of *Roseburia intestinalis* (*R. intestinalis*), the main species of the genus *Roseburia*, on the intestine [11, 12], we hypothesized that indigo exerts beneficial effects by acting as a prebiotic to enrich the commensal bacterium *R. intestinalis*. To test this hypothesis, we assessed the effects of indigo on *R. intestinalis* and found that indigo had a direct and significant growth‐promoting effect on the growth of *R. intestinalis in vitro* (Figure 1L). Next, mice were administered 2.5% DSS for 1 week, followed by *R. intestinalis* for 7 days (Figure S6A). Colonization of the mice with *R. intestinalis* resulted in a significant attenuation of intestinal inflammation (Figure 1M–O, Figure S6B–G) and improved the Th17/Treg balance (Figure 1P, Figure S6H–K). These *R. intestinalis*‐induced protective effects in colitis mice are consistent with the results described above, showing protection against intestinal inflammation in hosts treated with indigo or transferred indigo‐induced microbiota.

### Gut microbiota‐derived butyrate regulates the differentiation of naïve CD4^+^ T cells in vivo and in vitro

**Figure 2:**
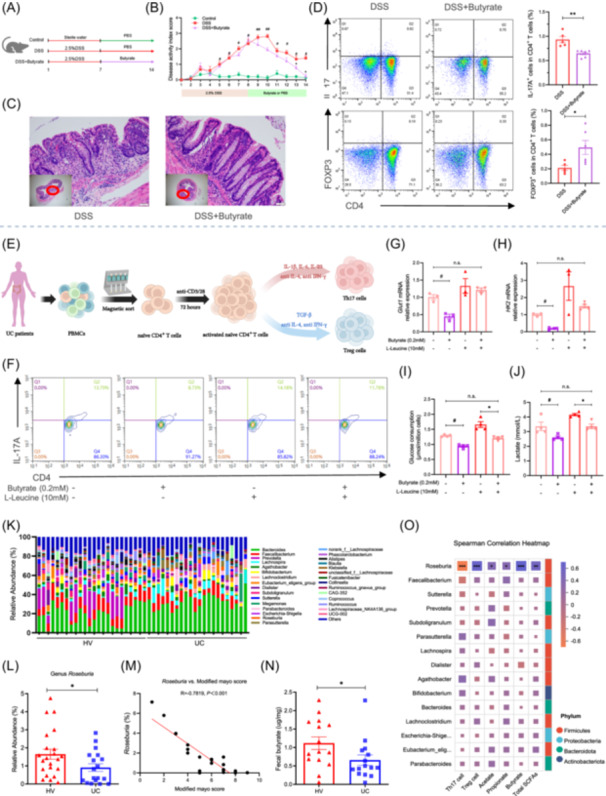
Gut microbiota‐derived butyrate regulates the differentiation of naïve CD4^+^ T cells in vivo and in vitro. To investigate the effect of butyrate on colitis in vivo, C57BL/6 mice were administered 2.5% DSS for 1 week, followed by 200 mM butyrate in drinking water, and the clinical phenotypes and the severity of colitis were assessed throughout the experiment. (A) Experimental schematic. (B) DAI score. (C) H&E staining (40× and 200× magnification) of colon tissues. MLNs from each mouse were collected for flow analysis. (D) Th17s and Tregs in MLNs from each group were analyzed by flow cytometry, and the results were displayed as bar charts. All data are presented as means ± SEM (*n* = 4–6 per group) from one of three experiments performed showing similar results. ^##^ *p* < 0.01, ^#^ *p* < 0.05 versus the Control group; ∗∗*p* < 0.01, ∗*p* < 0.05 versus the DSS group. (E, F) The differentiation of naïve CD4^+^ T cells into Th17 cells in the presence or absence of butyrate or mTORC1 agonist l‐leucine by flow cytometry. (G, H) The mRNA levels of Glut1 and HK2 in Th17‐polarizing CD4^+^ T cells were analyzed by RT‐qPCR. (I, J) The level of glucose in Th17‐cell lysate and lactate in supernatants were determined by spectrophotometer. All data are presented as mean ± SEM (*n* = 4 per group) from one of three experiments performed showing similar results. Human fecal specimens were collected from 24 healthy volunteers and 22 ulcerative colitis patients for bacterial 16S rRNA sequencing analysis, and all data are presented as mean ± SEM. (K) The composition of the bacterial microbiota in different groups at genus level. (L) The relative abundance of *Roseburia* was analyzed. (M) Spearman correlation analyses between the relative abundance of genus *Roseburia* and modified Mayo score in UC patients. (N) Human fecal specimens were collected for butyrate analysis by gas chromatography coupled with mass spectrometry. (O) Spearman correlation analyses between the relative abundance of the top 15 genera and Th17, Treg cell, or SCFAs levels in UC patients. ANOVA followed by Tukey's multiple comparison's test for B, J; Brown–Forsythe or Welch ANOVA tests for G, I; Kruskal–Wallis test followed by Dunn's multiple comparisons test for E; Student's *t*‐test for D (Tregs); and Mann–Whitney *U* test for D (Th17s), L, N. ∗∗*p* < 0.01, ∗*p* < 0.05 versus the HV group; n.s., not significant.

Our previous results showed significant enrichment of short‐chain fatty acid (SCFA)‐producing bacteria such as *Roseburia* and enhanced fatty acid biosynthesis and metabolism in indigo‐treated mice (Figure S7), suggesting that SCFAs might play a key role in the rebalance of immune homeostasis by indigo‐altered gut microbiota. A targeted metabolomic assay demonstrated that indigo markedly increased the levels of butyrate in colitis (Figure S8A–G), and Spearman correlation analyses showed a positive correlation between the relative abundance of *Roseburia* and butyrate levels in mice (Figure S8H). To further elucidate the role of butyrate in colitis, mice were administered 2.5% DSS, followed by 200 mM butyrate for 1 week, which significantly accelerated intestinal mucosal healing and improved the Th17/Treg balance (Figure 2A–D, Figure S8I–O). We then investigated the effects of butyrate on CD4^+^ T cell differentiation. Flow cytometry analysis revealed that butyrate significantly regulated the differentiation of naïve CD4^+^ T cells, as evidenced by the lower numbers of Th17 cells and higher numbers of Tregs in butyrate‐treated CD4^+^ T cells (Figure S9A–D). We further showed that butyrate significantly compensated expression of glycolysis‐related rate‐limiting enzymes in Th17 cells in the presence of butyrate, which corresponded with decreased levels of glucose consumption and lactate production (Figure S9E–J).

Mammalian target of rapamycin complex 1 (mTORC1) serves as a central regulatory factor in growth and metabolism [13]. The results from Figure S9K,L showed a significant decrease of Raptor (the main component of mTORC1) and HIF‐1α, a downstream protein of mTORC1 that translocates into the nucleus, enhances aerobic glycolysis, and controls CD4^+^ T cell fate [14, 15], in butyrate‐treated Th17 cells, suggesting that the mTORC1/HIF‐1α signal might be involved in differentiation of naïve CD4^+^ T cells. Then, we used l‐leucine, a selective mTORC1 agonist, to potentiate the mTORC1/HIF‐1α signal in vitro and showed that the immunosuppressive effect of butyrate was abolished in the presence of l‐leucine (Figure 2E,F, Figure S10A), accompanied by relieved expressions of glycolytic enzymes, glucose consumption, and lactate production (Figure 2G–J, Figure S10B,C). Taken together, these findings indicate that the mTORC1/HIF‐1α signal is involved in the differentiation of naïve CD4^+^ T cells induced by butyrate and may be a potential target for the treatment of UC.

### Relative abundance of genus *Roseburia* correlate with Th17/Treg cell and fecal butyrate level in UC patients

To determine whether the findings collected from animal models were similar to those from humans, we conducted a study to investigate the relationship between *Roseburia* Th17/Treg cells in UC patients. The results revealed that the overall observed OTUs and Shannon index in UC patients were significantly decreased (Figure S11A,B), and the gut microbiota structure differed significantly between the two groups (Figure S11C). Genus level analysis showed that the relative abundances of the harmful bacteria *Bacteroides* and *Lachnospira* were markedly increased, whereas the beneficial genus *Roseburia* was significantly decreased (Figure 2K,L). Spearman correlation analysis revealed a negative correlation between the genus *Roseburia* and the modified Mayo score, which is an important indicator of UC severity (Figure 2M). Moreover, we observed a marked imbalance of Th17/Treg cells in patients with UC (Figure S11D–G) and significantly decreased SCFAs, especially fecal butyrate level, in UC patients (Figure 2N, Figure S11H–J). Furthermore, the results from Spearman correlation analyses showed that the genus *Roseburia* was negatively correlated with Th17 cell numbers but positively correlated with Treg cell and fecal butyrate levels (Figure 2O), indicating a potential role in positively regulating intestinal inflammation.

## CONCLUSION

Our results demonstrate that orally administered indigo alleviates intestinal inflammation by promoting gut microbiota‐derived butyrate, which regulates CD4^+^ T cell differentiation and restores immune homeostasis, and holds promise as a novel microbial‐based immunotherapy for UC treatment.

## METHODS

The primer sequences used for RT‐qPCR and the baseline characteristics of the UC patients are listed in Tables S1 and S2. Detailed experimental materials and procedures, including sample collection and processing techniques, and statistical analysis, are provided in the Supplementary Material.

## AUTHOR CONTRIBUTIONS

**Yunqi Xing**: Data curation; formal analysis; investigation; validation; writing—original draft. **Muyuan Wang**: Data curation; formal analysis; investigation; visualization; writing—review and editing; methodology. **Yali Yuan**: Data curation; formal analysis; investigation; supervision; validation. **Jiayan Hu**: Data curation; supervision; formal analysis; validation; investigation. **Zhibin Wang**: Formal analysis; supervision; validation. **Zhongmei Sun**: Data curation; supervision; formal analysis; validation; investigation. **Mengyu Zheng**: Supervision; formal analysis; validation. **Lei Shi**: Supervision; formal analysis; validation. **Junxiang Li**: Project administration; supervision. **Tangyou Mao**: Conceptualization; data curation; formal analysis; funding acquisition; project administration; resources; supervision; writing—review and editing.

## CONFLICT OF INTEREST STATEMENT

The authors declare no conflicts of interest.

## ETHICS STATEMENT

All protocols were approved by the Institutional Review Board of Dongfang Hospital, Beijing University of Chinese Medicine (Nos. JDF‐IRB‐2017030802, JDF‐IRB‐2022031602), and the Animal Ethics Committee of Dongfang Hospital, Beijing University of Chinese Medicine (No. DFYY‐202104‐M). All patients and healthy subjects included in the study signed informed consent forms and agreed to participate in this study, as well as to the publication of related content.

## Supporting information


**Figure S1.** Administration of indigo ameliorated intestinal inflammation and improved Th17/Treg cell balance in DSS‐induced colitis mice.
**Figure S2.** Administration with indigo does not directly affect the differentiation of naïve CD4^+^ T cells sorted from UC patients into Th17/Treg cell.
**Figure S3.** The immunoprotective effects of indigo on intestinal inflammation in a gut microbiota‐dependent manner.
**Figure S4.** Altered microbiota of indigo‐treated colitis mice recapitulate the mucosal healing effects of indigo administration on intestinal inflammation.
**Figure S5.** Supplementation of indigo partially attenuates gut dysbiosis.
**Figure S6.** Indigo‐induced enrichment of *Roseburia intestinalis* alleviates intestinal inflammation in mice.
**Figure S7.** Microbial community functions predicted by PICRUSt using STAMP (version 2.1.3).
**Figure S8.** Gut microbiota‐derived butyrate ameliorates intestinal inflammation and restores Th17/Treg cells immune balance in DSS‐induced colitis.
**Figure S9.** Indigo‐induced enrichment of gut microbiota‐derived butyrate regulates the differentiation of naïve CD4^+^ T cells through glucose metabolism reprogramming.
**Figure S10.** mTORC1/HIF‐1α signal is involved in Th17 differentiation induced by butyrate in vitro.
**Figure S11.** The relative abundance of *Roseburia* correlates with Th17/Treg cell and fecal butyrate in UC patients.

**Table S1.** Primer Sequences Used for RT‐qPCR.
**Table S2.** Baseline Characteristics of the UC Patients.

## Data Availability

The data that supports the findings of this study are available in the supplementary material of this article. Data supporting the findings of this study are available in the Supplementary Material. The raw sequencing data from this study were deposited in the National Center of Biotechnology Information (NCBI) Sequence Read Archive (SRA) database under the BioProject accession numbers PRJNA1091305 (https://www.ncbi.nlm.nih.gov/bioproject/PRJNA1091305/) and PRJNA718382 (https://www.ncbi.nlm.nih.gov/bioproject/PRJNA718382/). The data and scripts used are saved in GitHub https://github.com/XYQ-0708/Indigo-micro. Supplementary materials (methods, figures, tables, graphical abstract, slides, videos, Chinese‐translated version, and updated materials) can be found in the online DOI or iMeta Science (http://www.imeta.science/).
