# Supplementary material for: Gut microbiota‐derived butyrate mediates the anticolitic effect of indigo supplementation through regulating CD4+ T cell differentiation
Source: Imeta. 2025 Apr 19;4(3):e70040. doi: 10.1002/imt2.70040 (PMC12130576; doi:10.1002/imt2.70040)
Supplement: Supplementary file 1 — Figure S1. Administration of indigo ameliorated intestinal inflammation and improved Th17/Treg cell balance in DSS‐induced colitis mice. Figure S2. Administration with indigo does not directly affect the differentiation of naïve CD4+ T cells sorted from UC patients into Th17/Treg cell. Figure S3. The immunoprotective effects of indigo on intestinal inflammation in a gut microbiota‐dependent manner. Figure S4. Altered microbiota of indigo‐treated colitis mice recapitulate the mucosal healing effects of indigo administration on intestinal inflammation. Figure S5. Supplementation of indigo partially attenuates gut dysbiosis. Figure S6. Indigo‐induced enrichment of Roseburia intestinalis alleviates intestinal inflammation in mice. Figure S7. Microbial community functions predicted by PICRUSt using STAMP (version 2.1.3). Figure S8. Gut microbiota‐derived butyrate ameliorates intestinal inflammation and restores Th17/Treg cells immune balance in DSS‐induced colitis. Figure S9. Indigo‐induced enrichment of gut microbiota‐derived butyrate regulates the differentiation of naïve CD4+ T cells through glucose metabolism reprogramming. Figure S10. mTORC1/HIF‐1α signal is involved in Th17 differentiation induced by butyrate in vitro. Figure S11. The relative abundance of Roseburia correlates with Th17/Treg cell and fecal butyrate in UC patients. [file IMT2-4-e70040-s002.docx]

**Supporting information to** **Gut microbiota-derived butyrate mediates the anticolitic effect of indigo supplementation through** **regulating CD4^+^ T cell differentiation**

**Running title**: Gut microbiota-derived butyrate mediates the anticolitic effect of indigo

Yunqi Xing^1,2#^, Muyuan Wang^1#^, Yali Yuan^1^, Jiayan Hu^1^, Zhibin Wang^1^, Zhongmei Sun^3^, Mengyu Zheng^4^, Lei Shi^1^, Junxiang Li^1*^, and Tangyou Mao^1*^

^1^ Dongfang Hospital, Beijing University of Chinese Medicine, Beijing, P.R. China. 100078.

^2^ Yueyang Hospital of Integrated Traditional Chinese and Western Medicine, Shanghai University of Traditional Chinese Medicine, Shanghai, P.R. China. 200437.

^3^ Tianjin Nankai Hospital, Tianjin, P.R. China. 300100.

^4^ King's College, London, UK. WC2R 2LS‌.

^#^These authors contributed equally: Yunqi Xing, Muyuan Wang

^*^Correspondence: maotangyouqun@126.com (Tangyou Mao); [lijunxiang1226@163.com](mailto:lijunxiang1226@163.com) (Junxiang Li)

# Supplementary Materials and Methods

**Preparation of indigo**

Indigo (synthetic, dye content 95 %, Sigma-Aldrich, St. Louis, MO, USA) was dissolved at 60 mg/kg or 300 mg/kg in phosphate buffered saline (PBS), sterilized through a 0.2-micron filter, and stored at 4℃ until use.

**Human fecal specimens**

Human fecal specimens were collected from 22 UC patients and 24 healthy individuals at the Department of Gastroenterology, Dongfang Hospital, Beijing University of Chinese Medicine. All participants provided written informed consent according to a protocol approved by the Institutional Review Board of Dongfang Hospital, Beijing University of Chinese Medicine (No. JDF-IRB-2017030802). All subjects were excluded for antibiotic or probiotic use in the last 2 months and during the sampling period. All stool samples were collected, sealed in aseptic containers, and stored at -80°C until further processing.

**Animals**

Wild-type (WT) female C57BL/6 mice (6-8 weeks of age, 18–20 g) were obtained from SPF Biotechnology Co., Ltd. (Beijing, China). In the process of the experiment, animals were fed food and sterile water ad libitum and kept in a specific-pathogen-free laboratory animal room on a 12 h/12 h light/dark cycle (22 ± 2℃ with a relative humidity of 50-60%) at Dongfang Hospital, Beijing University of Chinese Medicine (Beijing, China). All experimental protocols were approved by the Animal Ethics Committee of Beijing University of Chinese Medicine (No. BUCM-4-2019091603-3065) according to the guidelines issued by the Regulations of Beijing Laboratory Animal Management.

**DSS-induced colitis and drug treatment**

After 5 days of adaptive feeding, the mice were given free access to sterile water containing 2.5% DSS (w/v; 36,000-50,000 Da; MP Biomedicals, Santa Ana, CA, United States) for 1 week to induce colitis, followed by 7 days of intragastric administration of an indigo suspension at a dose of 60 mg/kg (DSS+ Indigo-L) or 300 mg/kg (DSS+ Indigo-H), or PBS (DSS). Mice in the control group were administered sterile water during the experimental period and were orally treated with the same volume of PBS from day 8. Each mouse was observed and clinical parameters were recorded daily, including stool consistency, rectal bleeding, and body weight. All mice were sacrificed on day 15.

**Antibiotic treatment**

The antibiotics were administered according to a previously published protocol. Briefly, after inducing colitis, the mice were treated with PBS indigo at a dose of 60 mg/kg in the absence or presence of broad-spectrum antibiotics in drinking water from Day8 to Day14. The antibiotic cocktail, a treatment regime that has been shown to effectively deplete the gut microbiota, consists of kanamycin (0.4 mg/ml), gentamicin (0.035 mg/ml), metronidazole (0.215 mg/ml), vancomycin (0.045 mg/ml), and colistin (850 U/ml). Growth performance and severity of colitis in all mice were evaluated daily.

**Fecal microbiota transplantation (FMT)**

The FMT experiment was performed according to a previously established method. Donor mice were randomly assigned into two groups, the DSS group and DSS+ Indigo group, and were administered 2.5% DSS for 7 days, followed by the administration of PBS or indigo at a dose of 60 mg/kg from day 8. Fecal material (stool pellets, and cecal and colonic contents) from donor mice were collected under a laminar flow hood in sterile condition once a day, resuspended in phosphate buffered saline (PBS) at 200 mg/ml and centrifuged at 2000 rpm for 1 min under 4℃. After induction of the colitis model with 2.5% DSS for 7 days, all recipient mice were gavaged once a day with 0.2 ml fecal slurry of stool pellets from the DSS group or DSS+ Indigo group mice. Their clinical status was assessed throughout the experiment, and the severity of colonic inflammation was scored after they were euthanized on day 15.

**Mouse intervention study with *Roseburia intestinalis***

C57BL/6 mice were given 2.5% DSS for 1 week followed by 1×10^9^ CFU *Roseburia intestinalis* (purchased from Bloomage Biotechnology Corporation Limited, China) suspended in 150 μl or PBS per day for 7 days. Growth performance and severity of colitis were evaluated in all mice. All mice were sacrificed on day 15.

**Butyrate administration in colitis mice *in vivo***

To investigate the effect of butyrate on colitis *in vivo*, C57BL/6 mice were administered 2.5% DSS for 1 week, followed by 200 mM butyrate (Sigma-Aldrich, Darmstadt, Germany) in their drinking water, and the clinical phenotypes and severity of colitis were assessed throughout the experiment.

***In vitro* bacterial cultures of *Roseburia* *intestinalis* with indigo treatment**

*R. intestinalis* was cultured in a modified reinforced clostridial medium at 37°C under strictly anaerobic conditions (100% N_2_), resuspended in modified reinforced clostridial medium or modified reinforced clostridial medium containing different concentrations of indigo (4 mg/mL and 0.4 mg/mL, respectively). The level of *R. intestinalis* was determined by measuring the bacterial density at 600 nm at 72 h using a microplate reader.

**Disease activity index (DAI)**

Body weight, stool consistency, and stool bleeding were recorded daily to determine the severity of colitis. The DAI was calculated as previously described.

**Reverse transcription quantitative polymerase chain reaction (RT-qPCR)**

Total RNA from homogenized mouse colonic tissues and CD4^+^ T cells sorted from UC patients were isolated using the TRIzol reagent (Invitrogen). Next, RT-qPCR reactions were conducted with One Step TB Green® PrimeScript™ RT-PCR Kit II (Perfect Real Time) (Takara) on the 7,300 Real Time PCR System (Applied Biosystems). The primer sequences used for RT-qPCR are listed in **Supplementary Table 1**. Relative genic quantification analysis was undertaken using the 2^-△△Ct^ method and normalized with GAPDH as a housekeeping control.

**Flow cytometry**

The spleens and mesenteric lymph nodes (MLNs) of mice were collected and stored in complete Dulbecco’s modified Eagle medium. The single cell suspensions were prepared by mechanically grinding with a nylon membrane (100 μm pore diameter), lysing with red blood cell lysis buffer and centrifugating at 2000 rpm for 5 min. Subsequently, the single cell suspensions were incubated with a cell stimulation cocktail (plus protein transport inhibitors) at 37°C in 5% CO2 for 6–8 h. Cells were stained for surface markers with APC/Cyanine7 anti-mouse CD3 antibody (BioLegend, Cat. No. 100222), and FITC anti-mouse CD4 antibody (BioLegend, Cat. No. 100509). Monocytes were fixed and permeabilized with fixation/permeabilization solution (BD Biosciences, CA, United States) before intracellular cytokine staining, and then antibodies including PerCP anti-mouse IL-17A antibody (BioLegend, Cat. No. 506944) and FOXP3 Monoclonal Antibody (FJK-16s) (eBioscience, Cat. No. 17-5773-82). The percentages of Th17 (CD3^+^ CD4^+^ IL17^+^) cells and Treg (CD3^+^ CD4^+^ FOXP3^+^) cells were analyzed using FlowJo V.10 software.

**16S rRNA sequencing analysis**

Fecal genomic DNA was extracted from 200 mg frozen samples using an E.Z.N.A. Soil DNA kit (Omega Bio-Tek, Norcross, GA, United States) according to the manufacturer’s protocols. This extraction method aligns with our previously established research protocols, as outlined in our previous study. DNA quality was determined by 1% agarose gel electrophoresis using a NanoDrop 2000 UV-vis spectrophotometer (Thermo Scientific, Wilmington, United States). PCR amplification of the V3~V4 hypervariable regions of the bacterial 16S rRNA gene was performed on a thermocycler PCR system (GeneAmp 9700, Applied Biosystems, Carlsbad, CA, United States) with the primers 338F: 5’-CCTACGGGNBGCASCAG-3’ and 806R: 5’-GACTACNVGGGTATCTAATCC-3’. All PCR products were purified using an AxyPrep DNA Gel Extraction Kit (Axygen Biosciences, Union City, CA, United States). Library preparation was performed using the NEXTflex Rapid DNA-Seq Kit (Bioo Scientific, Austin, TX, United States). Purified amplicon library was pooled at equal molar concentrations and paired-end sequenced on an Illumina MiSeq platform. Operational taxonomic units (OTUs) were generated and clustered at a 97% similarity threshold using Usearch (version 7.0). Bacterial alpha diversity was determined by sampling-based OTU analysis using Mothur (version 1.30.1) and presented by the Shannon index. Principal coordinate analysis (PCoA) was performed using representative sequences of OTUs for each sample according to the Bray–Curtis distance. Bacterial taxonomic analyses and comparisons, including bacterial phylum and genus levels, were conducted between the two groups using the Wilcoxon rank-sum test, which was visualized using R package (Version 2.15.3).

**Targeted measurement of fecal short-chain fatty acids (SCFAs)**

The levels of SCFAs in the feces were analyzed using gas chromatography coupled with mass spectrometry (TRACE 1300-ISQ 7000; Thermo Fisher Scientific, USA). 200 mg of feces were extracted in 50 μl of 15% phosphoric acid and 400 μl ether and centrifuged at 4°C for 10 min at 12000 rpm after vortexing for 60 s. The supernatant was preserved. Mixtures of seven SCFAs (acetic, propionic, isobutyric, butyric, isovaleric, valeric, and caproic acids) were used as standards and were purchased from Sigma-Aldrich (St. Louis, MO, USA). Helium was used as a carrier gas at a flow rate of 1 mL/min**.** The temperatures of the ion source and interface were 300°C and 250°C, respectively. The column temperature was programmed to increase from an initial temperature of 90°C, followed by an increase to 120°C at 10°C/min, to 150°C at 5°C/min, and finally to 250°C at 25°C/min, which was maintained for 2 min. The detector was operated in the electron impact ionization mode (electron energy 70 eV) using single-ion monitoring mode.

**Human naïve CD4^+^ T cell isolation**

Blood samples were obtained from patients (age 18-65 years) with mild to moderate UC diagnosed at least 3 months prior to enrollment at Dongfang Hospital, Beijing University of Chinese Medicine, following the acquisition of written informed consent. Peripheral blood mononuclear cells (PBMCs) were isolated from diluted blood samples through density-gradient centrifugation employing lymphocyte separation medium (Solarbio, Beijing, China). Subsequently, naïve CD4^+^ T cells were purified using the Human Naive CD4^+^ T Cell Isolation Kit II (Miltenyi Biotec, Shanghai, China). This research endeavor was granted approval by the Medical Ethics Committee of Dongfang Hospital, Beijing University of Chinese Medicine (Ethics Approval Number: JDF-IRB-2022031602) and has been registered with the Chinese Clinical Trial Registry under registration number ChiCTR2200063465, with a registration date of September 7, 2022.

***In Vitro* Th17 and Treg cell differentiation**

Naïve CD4^+^ T cells were cultivated in a 24-well plate (1 × 10^6^ cells/well) for three days in the presence of plate-bound anti-CD3 antibody (5 µg/mL, eBioscience^TM^) and anti-CD28 antibody (2 µg/mL, eBioscience^TM^). For the induction of Th17 cell differentiation, a cocktail of cytokines and antibodies including IL-1β (10 ng/mL, R&D), IL-6 (30ng/mL, R&D), IL-23 (20 ng/mL, R&D), anti-IFN-γ antibody (10 µg/ml, eBioscience^TM^), and anti-IL-4 antibody (10 µg/ml, eBioscience^TM^) were added. Alternatively, for the induction of Treg cell differentiation, TGF-β (10 ng/mL, R&D), anti-IFN-γ antibody (10 µg/ml, eBioscience^TM^), and anti-IL-4 antibody (10 µg/ml, eBioscience^TM^) were added. These cells were treated with butyrate at a dose of 0.2 mM or medium for 5 days. The cells underwent flow cytometry analysis, RT-qPCR assessment, and glucose consumption measurement. Additionally, the supernatants from these cells were harvested for enzyme-linked immunosorbent assay (ELISA) and lactate detection.

**Glucose consumption and lactate detection**

Glucose (Solarbio, BC2500), and lactate (Nanjing Jiancheng Bioengineering Institute A019-2-1) level in cell supernatant were determined by spectrophotometer according to the manufacturers’ instructions.

**Statistical analysis**

All data were represented as mean ± standard error of the mean (SEM), and were statistically analyzed and graphed using GraphPad Prism 8.0 (Graph Pad Software, La Jolla, CA, United States). The Shapiro–Wilk test was used to determine the sample distribution type. For two groups, the F test was used to test homoscedasticity, and significance was calculated by Two-tailed Student's *t*-test, Welch's *t*-test, or non-parametric Mann–Whitney *U* test. For more than two groups, the Brown-Forsythe test or Bartlett's test were used to test homoscedasticity, and the significance was calculated by one-way analysis of variance (ANOVA) followed by Tukey’s multiple comparison’s test, Brown-Forsythe and Welch tests, or the Kruskal–Wallis test followed by Dunn's multiple comparisons test. The correlation analysis was investigated by Spearman's rank correlation test. Results were considered to be statistically significant with *p* < 0.05.

# Supplementary Figures

## **Figure S1. Administration of indigo ameliorated intestinal inflammation and** improved Th17/Treg cell balance in DSS- induced colitis mice. C57BL/6 mice were free access to sterile water containing 2.5% DSS for 1 week to induce colitis followed by 7 days intragastric administration of indigo suspension at a dose of 60mg/kg and 300mg/kg or PBS. (A, B) Macroscopic image and measured length of the colon. (C) Spleen-weight ratio. (D) Representative photomicrographs of Hematoxylin and eosin (H&E) stained colon sections (40× and 200× magnification). (E-H) Representative plot and bar charts of the percentage of CD4^+^ IL17^+^ (Th17) cells and CD4^+^ FOXP3^+^ (Treg) cells in the spleen. (I, J) Colonic tissues were collected to detect the expression s of IL-17 and IL-10 by RT-qPCR analysis. All data are presented as mean ± SEM (*n* = 4-6 mice per group) from one of three experiments performed showing similar results. Brown-Forsythe or Welch ANOVA tests for B, C; Student's *t*-test for H, J; Welch's *t*-test for I; Mann-Whitney U test for G. ^#^*p* < 0.05 versus the Control group; ^**^*p* < 0.01, ^*^*p* < 0.05 versus the DSS group; n.s., not significant (*p* > 0.05).

## Figure S2. Administration with indigo does not directly affect the differentiation of naïve CD4^+^ T cells sorted from UC patients into Th17/Treg cell. Naïve CD4^+^ T cells were sorted from IBD patients, cultured under Th17- or Treg-polarizing conditions with butyrate at a dose of 0.2 mM or medium for 5 days (A). The purified naïve CD4^+^ T cells from UC patients were sorted by magnetic sort. (B, C, E, F) Under Th17- or Treg- polarizing conditions, the differentiation of naïve CD4^+^ T cells into Th17 or Treg cells in the presence or absence of butyrate by flow cytometry. (D, G) ELISA analysis for IL-17A and IL-10 in these different polarizing CD4^+^ T cells. All data are presented as mean ± SEM (*n* = 5 per group) from one of three experiments performed showing similar results. Student's *t*-test for C, D, F, G. *p* > 0.05, n.s., not significant.

## Figure S3. The immunoprotective effects of indigo on intestinal inflammation in a gut microbiota-dependent manner. Mice were given 2.5% DSS (w/v) in drinking water for 7 days followed by one week treatment of indigo suspension at a dose of 60mg/kg or PBS daily in the presence or absence of antibiotics, then the clinical parameters were recorded daily. (A) The experimental procedure. (B) The total DNA of gut microbiota in different groups after antibiotics treatment. (C) Drinking DSS volume per mouse. (D) Body weight. (E) Rectal bleeding score. (F) Stool consistency score. (G) DAI score. (H) Gross appearance of the colon from each group. (I, J) H&E staining (40× and 200× magnification) and histopathological scores of colon tissues. MLNs from each mouse were collected for flow analysis. (K, L) Th17 and Treg cells in the MLN from each group were analyzed by flow cytometry. All data are presented as mean ± SEM (*n* = 4-6 mice per group). ANOVA followed by Tukey’s multiple comparison’s test for B, C, D, E, F, G, K; Brown-Forsythe or Welch ANOVA tests for L; Kruskal-Wallis test followed by Dunn’s multiple comparisons test for J. ^#^*p* < 0.05, ^##^*p* < 0.01, versus the Control group; ^∗^*p* < 0.05 versus the DSS group; n.s., not significant (*p* > 0.05).

## Figure S4. Altered microbiota of indigo-treated colitis mice recapitulate the mucosal healing effects of indigo administration on intestinal inflammation. Fecal material (stool pellets, and cecal and colonic contents) from donor mice were collected and orally gavaged to the corresponding recipients. Clinical status was assessed throughout the experiment, and spleen, MLNs, colon from each mouse were collected for flow analysis and RT-qPCR. (A) The schematic diagram of FMT. (B) Daily DSS intake per mouse. (C) Rectal bleeding score. (D) Stool consistency score. (E) Colonic morphology. (F) Macroscopic appearance of the spleen. (G) Spleen-weight ratio. (H) Calculation of histopathological score of the colon. (I) Th17 cells in the spleen were analyzed by flow cytometry and bar charts of the proportion of Th17 cells were displayed. (J) Graph analysis of Treg cells in the spleen from each mouse. (K, L) Expressions of IL-17 and IL-10 mRNA were assessed by RT-qPCR. All data are presented as mean ± SEM (*n* = 5-6 mice per group) from one of two experiments performed showing similar results. ANOVA followed by Tukey’s multiple comparison’s test for B, C, D; Student's *t*-test for G, H, I, J, K, L. ^∗∗^*p* < 0.01, ^∗^*p* < 0.05 versus the DSS→DSS group.

## Figure S5. Supplementation of indigo partially attenuates gut dysbiosis. Fecal samples from all mice were collected for microbiome profile analysis by using bacterial 16S rRNA gene sequencing analysis. (A) Comparison of the observed bacterial species. (B-D) Alpha diversity represented by Shannon, ACE, and Chao index among different groups. (E) PCoA based on Bray-Curtis metric distances of beta diversity. (F) Relative abundance of bacteria at phylum level. (G) Linear discriminant analysis effect size (LEfSe) analysis to identify differentially abundant bacterial taxa among groups (linear discriminant analysis score > 2). All data are presented as mean ± SEM (*n* = 4-6 mice per group). ANOVA followed by Tukey’s multiple comparison’s test for A, B, C, D. ^#^*p* < 0.05 versus the Control group; ^∗^*p* < 0.05 versus the DSS group; n.s., not significant (*p* > 0.05).

## Figure S6. Indigo-induced enrichment of *Roseburia intestinalis* alleviates intestinal inflammation in mice. C57BL/6 mice were given 2.5% DSS for 1 week followed by 1×10^9^ CFU *Roseburia intestinalis* suspended in 150 ul or PBS per day for 7 days, then the growth performance and the severity of colitis of all mice were evaluated (A). (B) Daily DSS intake per mouse. (C, D) Macroscopic appearance of the spleen and spleen-weight ratio. (E) Colonic length. (F, G) Representative HE staining and calculation of histopathological score of the colon. (H, I) Th17 cells in spleen were analyzed by flow cytometry and bar charts of the proportion of Th17 cells were displayed. (J, K) Graph analysis of Treg cells in the spleen from each mouse. All data are presented as mean ± SEM (*n* = 5 mice per group) from one of two experiments performed showing similar results. Student's *t*-test for B, D, E, G, K; Welch's *t*-test for I. ^∗∗^*p* < 0.01, ^∗^*p* < 0.05 versus the DSS group.

## Figure S7. Microbial community functions predicted by PICRUSt using STAMP (version 2.1.3).

## Figure S8. Gut microbiota-derived butyrate ameliorates intestinal inflammation and restores Th17/Treg cells immune balance in DSS- induced colitis. (A-G) Fresh stool from all mice (5 mice/group) were collected from SCFAs analysis by gas chromatography coupled with mass spectrometry. (H) Spearman correlation analyses showed a positive correlation between the relative abundance of genus *Roseburia* and butyrate level in mice. For investigating the effect of butyrate on colitis *in vivo*, C57BL/6 mice were given 2.5% DSS for 1 week followed by 200 mM butyrate in drinking water, and the clinical phenotypes and the severity of colitis were assessed throughout the experiment. (I) Body weight. (J) Gross appearance of the colon. (K) Histopathological scores of colon tissues. Spleen from each mouse were collected for flow analysis. (L-O) Th17 and Treg cells in the spleen from each group were analyzed by flow cytometry. All data are presented as mean ± SEM (*n* = 5-6 mice per group) from one of two experiments performed showing similar results. ANOVA followed by Tukey’s multiple comparison’s test for B, C, D, E, F, I; Brown-Forsythe or Welch ANOVA tests for G; Kruskal-Wallis test followed by Dunn’s multiple comparisons test for A, K; Student's *t*-test for M, O. ^##^*p* < 0.01, ^#^*p* < 0.05 versus the Control group; ^∗∗^*p* < 0.01, ^∗^*p* < 0.05 versus the DSS group.

## Figure S9. Indigo-induced enrichment of gut microbiota-derived butyrate regulates the differentiation of naïve CD4^+^ T cells through glucose metabolism reprogramming. (A, B) Under Th17- or Treg- polarizing conditions, the differentiation of naïve CD4^+^ T cells into Th17 or Treg cells in the presence or absence of butyrate by flow cytometry. (C, D) ELISA analysis for IL-17A and IL-10 in these different polarizing CD4^+^ T cells. (E-H) The mRNA levels of Glut1, HK2, PKM2, and LDHA in Th17-polarizing CD4^+^ T cells were analyzed by RT-qPCR. (I, J) The level of glucose in Th17-cell lysate and lactate in supernatants were determined by spectrophotometer. (K, L) The mRNA levels of mTOR1 and HIF-1α were analyzed by RT-qPCR. All data are presented as mean ± SEM (*n* = 4-6 per group) from one of three experiments performed showing similar results. Student's *t*-test for A, B, C, D, E, F, G, H, I, J; Mann-Whitney U test for K, L. ^**^*p* < 0.01, ^*^*p* < 0.05 versus the Th17 group.

## Figure S10. mTORC1/HIF-1α signal is involved in Th17 differentiation induced by butyrate *in vitro*. (A) ELISA analysis for IL-17A in Th17-polarizing CD4^+^ T cells. (B, C) The mRNA levels of LDHA and HIF-1α in Th17-polarizing CD4^+^ T cells were analyzed by RT-qPCR. All data are presented as mean ± SEM (*n* = 4 per group) from one of three experiments performed showing similar results. ANOVA followed by Tukey’s multiple comparison’s test for A, B; Kruskal-Wallis test followed by Dunn’s multiple comparisons test for C. ^##^*p* < 0.01, ^#^*p* < 0.05 versus the Control group.

## Figure S11. The relative abundance of *Roseburia* correlates with Th17/Treg cell and fecal butyrate in UC patients. Human fecal specimens were collected from 24 healthy volunteers (HV) and 22 UC patients for bacterial 16S rRNA sequencing analysis. (A, B) Comparison of the observed bacterial species and Shannon index of different groups. (C) PCoA based on Bray-Curtis distance among different samples. (D-G) Th17 cell and Treg cell in PBMCs of HV (*n* = 15) and UC patients (*n* = 15) were determined by flow analysis. (H-J) Human fecal specimens were collected from 15 HV and 15 UC patients for SCFAs analysis by gas chromatography coupled with mass spectrometry. All data are presented as mean ± SEM. Student's *t*-test for B, J, L; Mann-Whitney U test for A, H, K, M.^∗∗^*p* < 0.01, ^∗^*p* < 0.05 versus the HV group; n.s. mean no statistical significance.
